# Association between leptin gene polymorphisms and hematological metabolic parameters in obese Saudi adults

**DOI:** 10.3389/fmed.2026.1841541

**Published:** 2026-06-05

**Authors:** Lamiaa Hamad Al-Jamea, Alexander Woodman, Rehab Yusuf Al-Ansari, Fatimah Salem Alayidh, Khalid Khalaf Alharbi, Nayef Saleh Al Ahmadi, Rashid Al-Jawair, Ibrahim Sahin, Shouq Saleh AlGhamdi, Emad Johar Al Johar, Jenifer Vecina Quiambao, Abdel Halim Deifalla, Yousef Mohammed Hawsawi

**Affiliations:** 1 Department of Scientific Research, King Fahad Military Medical Complex, Ministry of Defense Health Services, Dhahran, Saudi Arabia; 2 Adult Hematology Unit, Department of Internal Medicine, King Fahd Military Medical Complex, Dhahran, Saudi Arabia; 3 Department of Medical Laboratory, King Fahad Military Medical Complex, Ministry of Defense Health Services, Dhahran, Saudi Arabia; 4 Department of Clinical Laboratory Sciences, College of Applied Medical Sciences, King Saud University, Riyadh, Saudi Arabia; 5 Department of Academic Affairs and Training, King Fahad Military Medical Complex, Ministry of Defense Health Services, Dhahran, Saudi Arabia; 6 Department of Endocrinology, King Fahd Military Medical Complex, Dhahran, Saudi Arabia; 7 Al-Jawhara Centre for Molecular Medicine Genetics and Inherited Disorders, Arabian Gulf University, Manama, Bahrain; 8 Department of Anesthesia, Prince Sultan Military College of Health Sciences, Dammam, Saudi Arabia; 9 Graduate School, St. Paul University Manila, Manila, Philippines; 10 Department of Anatomy, Arabian Gulf University, Manama, Bahrain; 11 Research Center, King Faisal Specialist Hospital and Research Center, Jeddah, Saudi Arabia; 12 Department of Biochemistry and Molecular Medicine, College of Medicine, Alfaisal University, Riyadh, Saudi Arabia

**Keywords:** genetic analysis, hematological parameters, leptin gene, obesity, polymorphisms, variants

## Abstract

**Background:**

Obesity represents a major global public health challenge. However, among the Middle Eastern countries and members of the Gulf Cooperation Council, the Kingdom of Saudi Arabia (K.S.A) has been particularly affected, with prevalence rates exceeding 24.7%. The impact of obesity on hematological parameters has been previously established; nevertheless, the relationship between genetic variants and obesity-related hematological changes in the Saudi population remains unexplored.

**Aim:**

This study aimed to investigate the hematological and metabolic profiles of obese and non-obese Saudi subjects while determining potential associations between *LEP* single-nucleotide variants in the exon 1 5’-untranslated region (5’-UTR) and exon 2–3 coding regions.

**Methods:**

In the current study, we examined 202 Saudi adults (102 obese, 100 non-obese). Comprehensive hematological profiling was performed alongside genetic analysis of the leptin gene (*LEP*) through DNA sequencing of the exon 1 5’-UTR and exon 2–3 coding regions.

**Results:**

Obese subjects demonstrated significant alterations in hematological parameters compared to controls, including elevated white blood cell, platelet counts and decreased red blood cell counts. Red blood cell indices were significantly reduced in the obese group, including hemoglobin, hematocrit and mean corpuscular volume. These changes showed progressive alterations across obesity classes. Genetic analysis identified two *LEP* variants: NM_00230.2:c.-39G > A, which demonstrated significant association with obesity risk (OR: 2.48, 95% CI: 1.02-6.01, *p* = 0.04) and higher white blood cell counts, and c.280G > A, found exclusively in obese subjects (3.9%).

**Conclusion:**

This study reveals significant obesity-associated hematological alterations in the Saudi population, with progressive changes across obesity classes. The identification of *LEP* variants associated with obesity risk and altered white blood cell counts suggests potential genetic influences on obesity-related hematological changes in this population.

## 1 Introduction

Over the past years, obesity has represented a major global public health challenge (1). The condition is characterized by abnormal or excessive fat accumulation resulting from an imbalance between energy intake and expenditure, and has reached pandemic levels worldwide. Recent data indicate that obesity affects 878 million (16%) of the world’s adult population (2). This represents a significant global health challenge with profound implications for individual, public economic, and health outcomes (3). Among the Middle Eastern countries and members of the Gulf Cooperation Council, Saudi Arabia has been particularly affected, with a growing proportion of the adult population falling into the overweight and obese categories.

The Kingdom of Saudi Arabia (KSA) faces particularly concerning obesity trends, with recent studies reporting prevalence rates of 24.7%, according to national survey data, with the Eastern Region associated with the highest prevalence of 29.4% (4). Furthermore, according to the last census, Saudi Arabia had an estimated total population of 33.4 million, with around 56% being below the age of 30 years. In the year 2021, the Saudi government reported that 42.8% of the adult population was considered overweight or obese. Anticipated continuing growth of this trend and associated comorbidities, including type 2 diabetes and cardiovascular diseases, means that obesity remains a public health priority. This high prevalence has been attributed to rapid socioeconomic changes, urbanization, and adoption of Western lifestyles, characterized by decreased physical activity and increased consumption of energy-dense foods (5). This places a substantial burden on public health and healthcare systems, with estimates indicating an annual economic burden of $187 billion USD per capita per year between 2020 and 2050, representing ∼13% of health expenditure within KSA (6, 7).

The pathophysiology of obesity involves complex interactions between metabolic and hematological parameters. Adipose tissue functions as an active endocrine organ, releasing diverse adipokines including leptin, adiponectin, plasminogen activator inhibitor-1 (PAI-1) and pro-inflammatory cytokines such as tumor necrosis factor-α (TNF-α), interleukin-6 (IL-6), and monocyte chemoattractant protein-1 (MCP-1) that contribute to a chronic low-grade inflammatory state (8, 9). These changes manifest in elevated white blood cell counts, increased inflammatory markers, such as C-reactive protein and fibrinogen, and alterations in coagulation parameters, resulting in a state of hypercoagulation (10). These physiological changes contribute to several comorbidities, including insulin resistance, type 2 diabetes mellitus and cardiovascular diseases, collectively termed metabolic syndrome (11, 12).

The etiology of obesity encompasses a complex interplay of factors, including ethnic background, genetic predisposition (13), socio-economic status, lifestyle patterns, dietary habits, and physical activity levels (14). While environmental factors play a crucial role, growing evidence suggests a strong genetic component in obesity predisposition (15), epigenetic regulations and metabolomics (16), with genome-wide association studies identifying numerous genetic loci associated with body mass index (BMI) and body fat distribution (17–19).

Leptin, a peptide hormone produced by adipose tissue, forms part of a feedback mechanism to regulate food intake and energy expenditure (20). This feedback mechanism monitors the size of energy stores in the body, with altered expression occurring during pathological conditions, such as obesity. Genetic variation, expected to modulate the leptin feedback mechanism and circulating leptin levels, may affect the obesity traits of Saudi Arabian adults through specific gene polymorphisms. Several gene polymorphisms have been reported to be associated with diseases in the Saudi population, including breast cancer (21, 22), Colorectal Cancer (23), and iron deficiency status (15). Therefore, extensive research has established the significance of the leptin gene (*LEP*) and the leptin receptor gene (*LEPR*). Leptin, an adipocyte-derived peptide hormone, plays a crucial role in regulating food intake and energy expenditure through hypothalamic signaling pathways. More recently, in addition to obesity, it has been shown that abnormal metabolic and hematological profiles have emerged in Middle Eastern populations, including Saudis.

Variants in *LEP* and *LEPR* have been associated with autosomal recessive forms of obesity, leading to conditions such as congenital leptin deficiency (24–26). Although variants resulting in monogenic forms of obesity are rare, polymorphisms in *LEP* have shown variable associations with obesity across different populations (27–29). Of particular interest are findings from seven different cohort studies in Tunisia and Egypt, where eight obesity-associated *LEP* variants were identified, including two potentially pathogenic variants (c.104T > C and c.34delC) that appear to be exclusive to these populations, highlighting the potentially unique genetic makeup of this ethnic group (30–32).

While numerous studies have documented the impact of obesity on hematological and metabolic profiles, and studies have assessed associations between *LEP* variants and obesity in various populations, including the Arabic population, genetic research in the Saudi Arabian population remains limited (33). This knowledge gap is particularly significant given the growing obesity prevalence in this population. Therefore, this study aimed to investigate the hematological and metabolic profiles of obese and non-obese Saudi subjects while determining potential associations between *LEP* single-nucleotide variants in the exon 1 5’-untranslated region (5’-UTR) and exon 2–3 coding regions.

## 2 Materials and methods

### 2.1 Study design and eligibility criteria

The study was conducted at King Fahad Military Medical Complex (KFMMC) in the Eastern Province of Saudi Arabia. Obese (case) individuals were recruited from KFMMC outpatient clinics and non-obese (control) subjects from the KFMMC Blood Bank between May 1st, 2021 and February 27th, 2024.

Study participants were selected based on specific inclusion and exclusion criteria. Inclusion criteria encompassed subjects aged ≥ 18 years, those with a documented family history of obesity, and Saudi nationality. Exclusion criteria included subjects ≤ 18 years of age, those with BMI < 18.5 kg/m^2^, non-Saudi individuals, and subjects with medical conditions known to affect weight regulation. These conditions included thyroid disease, Cushing’s syndrome, polycystic ovarian syndrome, insomnia, congestive heart failure, and acromegaly. Additionally, subjects using medications that could influence weight, such as steroids and antidiabetic drugs. After application of the eligibility criteria, the final study sample comprised 102 obese and 100 non-obese subjects.

### 2.2 Ethical approval

Ethical approval was obtained from the Arabian Gulf University in the Kingdom of Bahrain (IRB No. E02-PI-11/19) and the Ethics Committee of Armed Forces Hospital in the Eastern Region, Kingdom of Saudi Arabia (AFHER-IRB-REN-2022-005). All participants provided written informed consent before enrollment.

### 2.3 Data collection

Anthropometric measurements, including height (cm) and weight (kg) were collected from obese and control individuals. All measurements were performed in duplicate, and the mean value was used for analysis.

Body mass index (BMI) was calculated as weight in kilograms divided by height in meters squared (kg/m^2^). Participants were classified according to World Health Organization (WHO) international BMI cut-off points, with underweight defined as < 18.5 kg/m^2^, normal weight as 18.5–24.9 kg/m^2^, overweight as 25.0–29.9 kg/m^2^, obesity class I as 30.0–34.9 kg/m^2^, obesity class II as 35.0–39.9 kg/m^2^, and obesity class III as ≥ 40.0 kg/m^2^.

### 2.4 Blood sampling

Three blood samples were collected by venipuncture for each enrolled individual after 12–14 h of overnight fasting. One sample (5 mL) was collected in a BD Vacutainer Serum tube (BD, Riyadh, Saudi Arabia) and two 5 mL samples were collected in an ethylenediaminetetraacetic acid (EDTA) tubes. The serum sample was used to estimate cholesterol, low-density lipoprotein (LDL), high-density lipoprotein (HDL), as well as triglyceride and glucose levels. The first EDTA sample was used for analysis of hematological parameters and the second for the extraction of genomic DNA.

### 2.5 DNA extraction and sequencing

Genomic DNA was extracted from peripheral EDTA blood samples using the QIAMP DSP DNA kit (Qiagen, Hilden, Germany), following the manufacturer’s instructions. Extracted DNA concentration and purity was determined using the Nanodrop-1000 spectrophotometer (ThermoFisher Scientific, Waltham, MA, United States). Samples with 260/280 ratios between 1.8 and 2.0 were considered acceptable for analysis.

PCR amplification was conducted for *LEP* exon 1 (5’-UTR), exon 2 and exon 3 regions using a standardized reaction mixture. Each 25μL reaction contained DreamTaq Green PCR Master Mix (12.5 μL at 2x concentrate) (ThermoFisher Scientific, Waltham, MA, United States), forward and reverse primers (1 μL each at 10 pmol/μL), template DNA (2μL at 50 ng/μL), and nuclease-free water (8.5 μL). The master mix provided DreamTaq DNA polymerase (1.25 units), optimized DreamTaq buffer, MgCl_2_ (2.0 mM), and dNTPs (0.2 mM each). Primer sequences and annealing temperatures are provided in Table 1.

**TABLE 1:** PCR amplification primer information and annealing temperatures.

Target LEP region	Primer sequence (5’–3’)	PCR Product size (bp)	Annealing temperature (°C)
Exon 1 (5’UTR)	**CCCCGCGAGGTGCACACTG** **GAGCGCGCCGGGGCCTTAC**	**213**	**56**
Exon 2	GTCTGGTAATGTGGTTGGTAAT TTCAGGAGGCGTTCAATAAATG	392	50
Exon 3	GCAGTCAGTCTCCTCCAA GTCCTGGATAAGGGGTGT	469	48

The thermal cycling conditions incorporated initial denaturation at 94°C for 5 min, followed by 35 amplification cycles. Each cycle consisted of denaturation at 94°C for 15 s, primer annealing for 30 s at primer-specific temperatures, and extension at 72°C for 30 s. The protocol concluded with a final extension step at 72°C for 5 min. No template (negative) controls were included in each PCR run.

PCR products were verified by agarose gel electrophoresis on 1.5% agarose before purification using the QIAquick PCR Purification Kit (Qiagen, Hilden, Germany). DNA sequencing was performed using the BigDye Terminator V3.1 Cycle Sequencing kit (ThermoFisher Scientific, Waltham, MA, United States) on an ABI 3730 (ThermoFisher Scientific, Waltham, MA, United States) sequencer. All samples were sequenced in both forward and reverse directions.

### 2.6 DNA sequencing analysis

The Matched Annotation from NCBI and EMBL-EBI (MANE) Select (34), NM_00230.2 *LEP* reference sequence was used for DNA sequence analysis. DNA sequencing chromatograms were aligned with the *LEP* reference sequence using InnoviGene Suite software (ThermoFisher Scientific, Waltham, MA, United States). Sequence quality was assessed using Phred quality scores, with scores > 20 considered acceptable. Variants in *LEP* were identified and listed in accordance with their corresponding positions on the reference genome. Human Genome Variation Society Nomenclature was utilized for description of variants (35). All variants were confirmed by independent analysis of forward and reverse sequences.

### 2.7 Statistical analysis

Statistical Package for the Social Sciences (SPSS version 30, Armonk, NY: IBM Corp., United States) was used to compile and analyze the acquired data. Differences in clinical variables (BMI, hematological and metabolic profiles) between cases and controls were evaluated using the non-parametric Mann-Whitney U test. These are presented as the mean ± standard deviation.

A comparison of genetic variants and other categorical variables between the two groups were compared using the Pearson’s Chi-square test, presented as frequencies and percentages. Genetic variants were analyzed for Hardy-Weinberg equilibrium. Odds ratios (OR) represent the association with confidence intervals (CI) at 95%. A *p* < 0.05 was considered to indicate statistical significance.

## 3 Results

### 3.1 Demographic and anthropometric characteristics

The study population comprised 202 participants, with 100 individuals in the control group and 102 in the obese group. Notable demographic differences were observed between the groups, shown in Table 2, with the obese cohort being significantly older (39.8 ± 11.3 vs. 32.6 ± 7.8 years, *p* < 0.0001) and having different gender distributions. While the control group was predominantly male (96/4 M/F ratio), the obese group showed a female predominance (35/67 M/F ratio). As expected, BMI was significantly higher in the obese group compared to the control group (34.4 ± 5.4 vs. 25.4 ± 2.9 kg/m^2^, *p* < 0.0001). Importantly, given the known physiological differences between males and females—particularly in hematological parameters—this imbalance represents a potential confounding factor.

**TABLE 2:** Anthropometric, hematological and metabolic characteristics of the control and obese study populations.

	Control group	Obese group	*p* value
*n* =	100	102	**< 0.0001**
Age	32.6 ± 7.8	39.8 ± 11.3	
Gender (M/F)	(96/4)	(35/67)	**< 0.0001**
BMI (kg/m^2^)	25.4 ± 2.9	34.4 ± 5.4	
Hematological parameters			
White blood cells (x10^9^/L)	6.1 ± 1.8	7.1 ± 3.2	**0.017**
Red blood cells (x10^12^/L)	5.3 ± 0.6	4.8 ± 0.8	**< 0.0001**
Hemoglobin (g/dL)	15.2 ± 1.3	12.6 ± 2.5	**< 0.0001**
Haemocrit (%)	46.3 ± 3.9	38.6 ± 7.2	**< 0.0001**
Mean corpuscular volume (fL)	88.1 ± 7.9	80.6 ± 11.8	**< 0.0001**
Mean cell hemoglobin (pg)	28.9 ± 2.8	26.3 ± 4.1	**< 0.0001**
Mean cell hemoglobin concentration (g/dL)	32.8 ± 1.0	32.6 ± 1.2	0.419
Red blood cell distribution width (%)	12.7 ± 0.9	14.0 ± 2.4	**< 0.0001**
Platelet (x10^9^/L)	248.4 ± 55.5	299.0 ± 136.5	**0.0001**
Metabolic parameters			
Glucose (mmol/L)	5.0 ± 4.9	6.3 ± 2.4	**< 0.0001**
Total Cholesterol (mmol/L)	5.2 ± 1.2	4.9 ± 1.0	0.058
Triglycerides (mmol/L)	1.5 ± 0.8	1.5 ± 1.2	0.319
LDL (mmol/L)	3.4 ± 0.9	3.0 ± 0.8	**0.032**
HDL (mmol/L)	1.4 ± 0.4	1.3 ± 0.3	**0.004**

Data is shown as the mean ± standard deviation. *p*-values were calculated using Mann Whitney U test, with statistical significance achieved if *p* < 0.05. BMI, body mass index; LDL, low density lipoprotein; HDL, high density lipoprotein.

### 3.2 Hematological and metabolic parameters in control and obese populations

Hematological parameters showed consistent differences between the groups, outlined in Table 2. The obese group demonstrated significantly elevated white blood cell (WBC) counts (7.1 ± 3.2 vs. 6.1 ± 1.8 × 10^9^/L, *p* = 0.017) and platelet counts (299.0 ± 136.5 vs. 248.4 ± 55.5 × 10^9^/L, *p* = 0.0001). In addition, a number of red blood cell (RBC) parameters were lower in the obese group, with significantly reduced RBC count (4.8 ± 0.8 vs. 5.3 ± 0.6 × 10^9^/L, *p* < 0.0001), hemoglobin (12.6 ± 2.5 vs. 15.2 ± 1.3 g/dL, *p* < 0.0001), and hematocrit (38.6 ± 7.2 vs. 46.3 ± 3.9%, *p* < 0.0001) observed. RBC indices relating to the size and hemoglobin content showed significant differences, with lower mean corpuscular volume (MCV) (80.6 ± 11.8 vs. 88.1 ± 7.9 fL, *p* < 0.0001) and mean corpuscular hemoglobin (MCH) (26.3 ± 4.1 vs. 28.9 ± 2.8 pg, *p* < 0.0001) in the obese group, while the mean corpuscular hemoglobin concentration (MCHC) remained similar between groups (*p* = 0.419). The red cell distribution width was significantly higher in the obese group (14.0 ± 2.4 vs. 12.7 ± 0.9%, *p* < 0.0001), indicating increased variation in RBC size in the obese group, compared to controls. Hemoglobin differences may reflect sex-related physiological variation rather than obesity alone.

Analysis of metabolic parameters revealed that the obese group had significantly higher glucose levels (6.3 ± 2.4 vs. 5.0 ± 4.9 mmol/L, *p* < 0.0001). Lipid profiles showed the obese group to have lower low-density lipoprotein (LDL) (3.0 ± 0.8 vs. 3.4 ± 0.9 mmol/L, *p* = 0.032) and high-density lipoprotein (HDL) (1.3 ± 0.3 vs. 1.4 ± 0.4 mmol/L, *p* = 0.004). Total cholesterol and triglycerides were not significantly different between groups (*p* = 0.058 and *p* = 0.319, respectively).

### 3.3 Comparative hematological parameter analysis across obesity classes

Further analysis stratified by WHO obesity classes (class I: 30.0–34.9 kg/m^2^, class II: 35.0–39.9 kg/m^2^, class III: ≥ 40.0 kg/m^2^) revealed progressive alterations in hematological parameters with increasing BMI (Table 3). Among the obese cohort, there were 56 class I obese individuals (54.9%), 27 class II (26.5%) and 19 class III (18.6%). Hemoglobin levels demonstrated a decrease across obesity classes (Type I: 12.8 ± 2.6 g/dL, Type II: 12.7 ± 1.9 g/dL, Type III: 11.9 ± 2.8 g/dL), with Type III showing significant differences compared to both Type I and II (*p* < 0.05). WBC counts increased with obesity severity (Type I: 6.7 ± 3.4 × 10^9^/L, Type II: 6.7 ± 2.0 × 10^9^/L, Type III: 7.9 ± 2.3 × 10^9^/L), while RDW showed progressive elevation trend with increasing obesity class (Type I: 13.7 ± 2.1%, Type II: 13.9 ± 2.1%, Type III: 14.8 ± 3.4%). MCHC was comparable across Type I and II but showed significant reduction in Type III obesity (32.0 ± 1.4 g/dL, *p* < 0.05 vs. control) (Table 3).

**TABLE 3:** Anthropometric, hematological and metabolic characteristics of the control and obese class type.

	Control group	Obese group		
		Type I	Type II	Type III
*n* =	100	56	27	19
Age	32.6 ± 7.8	39.6 ± 11.2	40.0 ± 11.7	39.7 ± 12.1
Gender (M/F)	(96/4)	(25/31)	(7/20)	(3/16)
BMI (kg/m^2^)	25.4 ± 2.9	32.4 ± 1.5*	36.9 ± 1.4*^†^	44.7 ± 6.4*^†#^
Hematological parameters				
WBC	6.1 ± 1.8	6.7 ± 3.4*	6.7 ± 2.0*	7.9 ± 2.3*
RBC	5.3 ± 0.6	5.0 ± 0.8*	4.7 ± 0.6*	4.7 ± 0.8*
Hemoglobin	15.2 ± 1.3	12.8 ± 2.6*	12.7 ± 1.9*	11.9 ± 2.8*^† #^
HCT	46.3 ± 3.9	39.2 ± 7.5*	38.6 ± 5.7*	37.3 ± 8.2*
Mean corpuscular volume	88.1 ± 7.9	80.0 ± 12.1*	82.7 ± 10.0*	80.4 ± 13.5*
MCH	28.9 ± 2.8	26.2 ± 4.3*	27.1 ± 3.3*	25.8 ± 4.6*
MCHC	32.8 ± 1.0	32.7 ± 1.1	32.8 ± 0.9	32.0 ± 1.4*
RDW	12.7 ± 0.9	13.7 ± 2.1*	13.9 ± 2.1*	14.8 ± 3.4*
Platelet	248.4 ± 55.5	288.4 ± 107.7*	287.3 ± 69.4*	284.8 ± 82.1*
Metabolic parameters				
Glucose (mmol/L)	5.0 ± 4.9	6.0 ± 1.5	6.0 ± 1.3	7.1 ± 4.1
Total cholesterol (mmol/L)	5.2 ± 1.2	4.9 ± 1.1	4.8 ± 0.8	4.9 ± 1.2
Triglycerides (mmol/L)	1.5 ± 0.8	1.5 ± 1.4	1.5 ± 1.1	1.5 ± 0.6
LDL (mmol/L)	3.4 ± 0.9	3.0 ± 0.8	3.0 ± 0.6	2.9 ± 1.0
HDL (mmol/L)	1.4 ± 0.4	1.3 ± 0.4	1.3 ± 0.3	1.1 ± 0.2*^†^

*p*-values were calculated using Mann Whitney U test, *statistical significance reached compared to control;

†statistical significance reached compared to type I; #statistical significance reached compared to type II. Statistical significance if *p* < 0.05.

### 3.4 Genetic analysis of the LEP gene

Sequence analysis of the coding region of *LEP* in the control and obese samples identified two variants, present in both heterozygous and homozygous forms. The first was a guanine to adenine single nucleotide transversion, NM_00230.2:c.-39G > A (historically G19A, rs2167270) within exon 1 of the 5’-UTR region. The second variant was a guanine to adenine single nucleotide transversion, within exon 3 of *LEP*, NM_00230.2:c.280G > A (historically G280A, rs17151919). This was predicted to result in the substitution of a valine amino acid with methionine in the leptin protein at position 94, p. (Val94Met).

### 3.5 Distribution of genotype and allelic frequency for the LEP c.-36G > A variant

In the presented results for the rs2167270 polymorphism (NM_00230.2:c.-39G > A), we observed genotype frequencies in the overall study population were: GG: 82.3%, GA: 12.9%, AA: 4.8%; expected Hardy–Weinberg equilibrium (HWE) frequencies were: GG: 78.76%, GA: 19.97%, AA: 1.27%; *p*-value = [(> 0.05], indicating no significant deviation from HWE.

Stratified analysis showed that in the control group, genotype frequencies were GG: 87.0%, GA: 8.0%, and AA: 5.0%, whereas in the obese group, they were GG: 77.5%, GA: 17.6%, and AA: 4.9%. The heterozygous GA genotype was associated with an increased risk of obesity compared to the GG genotype (OR: 2.48, 95% CI: 1.02–6.01, *p* = 0.04). The AA genotype showed no significant association (OR: 1.10, 95% CI: 0.31–3.95, *p* = 0.88). Under the dominant model (GA + AA vs. GG), a non-significant trend toward increased obesity risk was observed (OR: 1.95, 95% CI: 0.92–4.10, *p* = 0.08). Allelic analysis indicated a higher frequency of the A allele in obese individuals (13.7%) compared to controls (9.0%), although this did not reach statistical significance (OR: 1.61, 95% CI: 0.86–3.01, *p* = 0.13).

**FIGURE 1:**
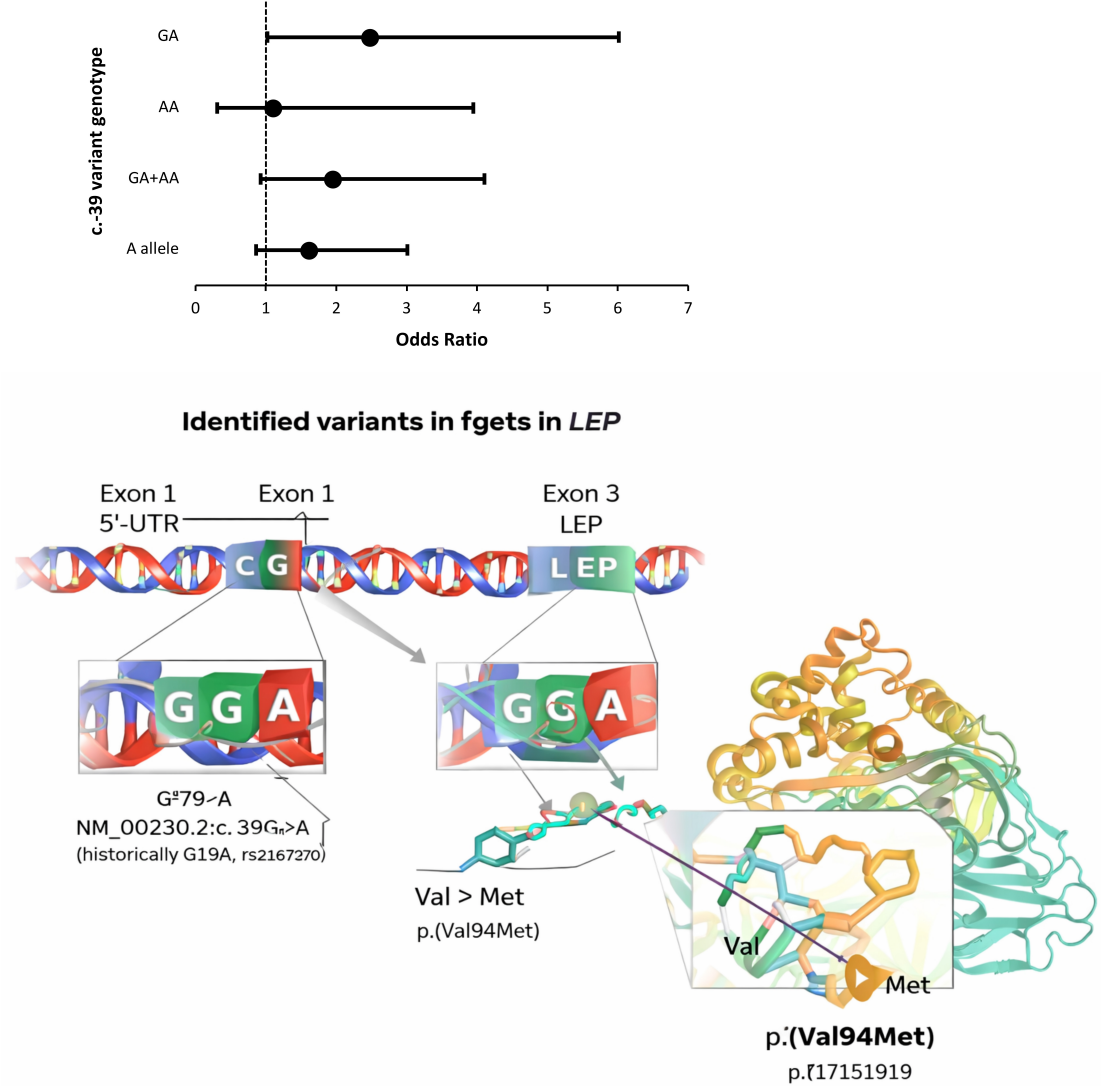
Odds ratios—c.-36G > A variant genotype.

On the other hand, for the rs7799039 polymorphism (c.280G > A) we observed genotype frequencies were: TT (GG): 98.0%, TC (GA): 2.0%, CC (AA): 0%; expected HWE frequencies were: TT: 98.01%, TC: 1.98%, CC: 0.01%; *p*-value = [(> 0.05], indicating no significant deviation from HWE (Figure 1 and Table 4).

**TABLE 4:** Genotype and allelic frequency distributions of the control and obese group populations in relation to the c.-36G > A variant genotypes.

Single nucleotide variant	Genotype	Control (%)	Obese (%)	Odds ratio (95% CI)	*p*-value
	Genotype frequency				
c.-39G > A (rs2167270)	GG	87 (87.0)	79 (77.5)	1	
	GA	8 (8.0)	18 (17.6)	2.48 (1.02–6.01)	0.04
	AA	5 (5.0)	5 (4.9)	1.10 (0.31–3.95)	0.88
	GA + AA	13 (13.0)	23 (22.5)	1.95 (0.92–4.10)	0.08
	Allelic frequency				
	G allele	182 (91.0)	176 (86.3)	1	
	A allele	18 (9.0)	28 (13.7)	1.61 (0.86–3.01)	0.13
c.280	Genotype frequency				
	GG	100 (100)	98 (96.1)	–	–
	GA	0 (0)	4 (3.9)	–	–
	AA	0 (0)	0 (0)	–	–

*p*-values were calculated using Pearson’s X^2^ test, based on comparison with homozygous *LEP* reference sequence (GG) for genotype or G for allelic frequency. Reference sequence: NM_000230.3(LEP). Statistical significance if *p* < 0.05.

### 3.6 Distribution of genotype and allelic frequency for the LEP c.280G > A variant

The NM_00230.2:c.280G > A, p.(Val94Met) (rs17151919) variant was only identified in the obese group, as outlined in Table 4. The variant was present in four obese subjects (3.9%) in the heterozygous form (GA), with the homozygote reference (GG) presenting in the remaining 98 subjects (96.1%).

### 3.7 LEP Genotype-phenotype correlations

A statistically significant elevation in WBC count was observed in obese subjects carrying the GA genotype of c.-39G > A variant (8.9 ± 5.4) compared to the GG reference genotype (6.6 ± 2.4, *p* < 0.05) (Table 5). This finding was further supported in the dominant model analysis (Table 6), where the combined GA + AA genotypes showed significantly higher WBC counts (8.6 ± 4.8) compared to the GG genotype in the obese group. All other hematological and metabolic parameters showed no statistically significant differences across the c.-39G > A or c.280G > A genotypes in either group.

**TABLE 5:** Hematological and metabolic characteristics of control and obese group populations in relation to the c.-36G > A and c.280G > A variant genotypes.

	Control group			Obese group			Obese group		
	c.-39G > A (rs2167270)						c.280 G > A (rs17151919)		
	GG	GA	AA	GG	GA	AA	GG	GA	AA
*n* =	87	8	5	79	18	5	98	4	–
Age	32.7 ± 7.7	33.8 ± 9.8	29.8 ± 6.4	39.9 ± 11.9	39.0 ± 9.2	40.6 ± 9.6	39.8 ± 11.4	42.0 ± 8.8	–
Gender (M/F)	(84/3)	(7/1)	(5/0)	(28/51)	(5/13)	(2/3)	(33/65)	(2/2)	–
BMI (kg/m^2^)	25.2 ± 3.0	27.1 ± 1.8	25.8 ± 0.9	35.7 ± 5.6	35.9 ± 4.8	36.1 ± 4.6	38.4 ± 20.8	38.6 ± 7.6	–
Hematological parameters									
WBC	5.9 ± 1.7	6.2 ± 1.8	7.2 ± 2.3	6.6 ± 2.4	8.9 ± 5.4*	7.4 ± 1.9	6.8 ± 2.5	6.1 ± 1.8	–
RBC	5.3 ± 0.6	5.4 ± 0.5	4.9 ± 0.4	4.9 ± 0.8	4.6 ± 0.7	4.8 ± 1.0	4.8 ± 0.8	5.3 ± 0.6	–
Hemoglobin	15.2 ± 1.3	15.2 ± 1.5	15.0 ± 1.2	12.6 ± 2.4	12.2 ± 2.6	13.4 ± 3.4	12.6 ± 2.5	15.2 ± 1.3	–
HCT	46.4 ± 3.9	46.5 ± 4.4	45.8 ± 2.9	38.8 ± 7.0	36.9 ± 7.5	40.9 ± 9.9	38.6 ± 7.2	46.3 ± 3.8	–
Mean corpuscular volume	87.9 ± 7.7	86.9 ± 9.7	93.8 ± 8.2	80.3 ± 11.8	80.8 ± 11.9	85.0 ± 11.6	80.5 ± 11.9	88.1 ± 7.9	–
MCH	28.9 ± 2.7	28.5 ± 3.6	30.7 ± 2.6	26.2 ± 4.1	26.7 ± 4.5	27.7 ± 3.7	26.3 ± 4.2	28.9 ± 2.8	–
MCHC	32.8 ± 1.0	32.7 ± 1.5	32.7 ± 1.1	32.5 ± 1.0	32.9 ± 1.8	32.6 ± 1.0	32.6 ± 1.2	32.8 ± 1.0	–
RDW	12.7 ± 0.9	12.5 ± 1.0	12.4 ± 0.6	14.0 ± 2.5	13.8 ± 2.4	13.6 ± 0.7	14.0 ± 2.5	12.7 ± 0.9	–
Platelet	249.4 ± 56.0	246.6 ± 62.7	234.4 ± 39.7	286.9 ± 91.5	305.7 ± 116.8	267.8 ± 68.0	299.2 ± 138.7	248.4 ± 55.5	–
Metabolic parameters									
Glucose (mmol/L)	5.2 ± 5.3	3.7 ± 1.2	5.0 ± 3.4	6.3 ± 2.4	6.5 ± 2.7	6.2 ± 1.1	6.3 ± 2.4	5.0 ± 5.0	–
Total cholesterol (mmol/L)	5.3 ± 1.1	5.2 ± 1.3	4.4 ± 0.8	4.8 ± 1.1	4.9 ± 0.9	4.9 ± 1.5	4.9 ± 1.0	5.2 ± 1.1	–
Triglycerides (mmol/L)	1.5 ± 0.8	1.8 ± 1.2	1.1 ± 0.5	1.5 ± 1.3	1.5 ± 0.8	1.2 ± 0.6	1.5 ± 1.2	1.5 ± 0.8	–
LDL (mmol/L)	3.4 ± 1.0	3.1 ± 0.7	2.6 ± 0.6	3.0 ± 0.8	2.9 ± 0.8	3.4 ± 1.2	3.0 ± 0.8	3.3 ± 0.9	–
HDL (mmol/L)	1.4 ± 0.3	1.58 ± 0.7	1.5 ± 0.3	1.3 ± 0.4	1.2 ± 0.3	1.2 ± 0.4	1.3 ± 0.8	1.4 ± 0.4	–

*p*-values were calculated using Mann Whitney U test, based on comparison with homozygous *LEP* reference sequence (GG). Reference sequence: NM_000230.3(LEP).

*Statistical significance if *p* < 0.05.

**TABLE 6:** Hematological and metabolic characteristics of control and obese group populations in relation to the c.-36G > A variant genotype (dominant model).

	Control group		Obese group	
	c.-39G > A (rs2167270)			
	GG	GA + AA	GG	GA + AA
*n* =	87	13	79	23
Age	32.7 ± 7.7	32.2 ± 8.6	39.9 ± 11.9	39.4 ± 9.1
Gender (M/F)	(84/3)	(12/1)	(28/51)	(7/16)
BMI (kg/m^2^)	25.2 ± 3.0	26.6 ± 1.6	35.7 ± 5.6	35.9 ± 4.7
Hematological parameters				
WBC	5.9 ± 1.7	6.9 ± 2.3	6.6 ± 2.4	8.6 ± 4.8*
RBC	5.3 ± 0.6	5.2 ± 0.5	4.9 ± 0.8	4.6 ± 0.8
Hemoglobin	15.2 ± 1.3	15.1 ± 1.3	12.6 ± 2.4	12.4 ± 2.8
HCT	46.4 ± 3.9	46.2 ± 3.7	38.8 ± 7.0	37.8 ± 8.0
Mean corpuscular volume	87.9 ± 7.7	89.6 ± 9.5	80.3 ± 11.8	81.7 ± 11.7
MCH	28.9 ± 2.7	29.3 ± 3.4	26.2 ± 4.1	26.9 ± 4.3
MCHC	32.8 ± 1.0	32.7 ± 1.3	32.5 ± 1.0	32.9 ± 1.7
RDW	12.7 ± 0.9	12.4 ± 0.8	14.0 ± 2.5	13.8 ± 2.2
Platelet	249.4 ± 56.0	241.9 ± 53.5	286.9 ± 91.5	339.9 ± 230.5
Metabolic parameters				
Glucose (mmol/L)	5.2 ± 5.3	4.2 ± 2.3	6.3 ± 2.4	6.4 ± 2.4
Total cholesterol (mmol/L)	5.3 ± 1.1	4.9 ± 1.2	4.8 ± 1.1	4.9 ± 1.0
Triglycerides (mmol/L)	1.5 ± 0.8	1.6 ± 1.0	1.5 ± 1.3	1.5 ± 0.7
LDL (mmol/L)	3.4 ± 1.0	2.9 ± 0.7	3.0 ± 0.8	3.0 ± 0.8
HDL (mmol/L)	1.4 ± 0.3	1.5 ± 0.6	1.3 ± 0.4	1.2 ± 0.3

*p*-values were calculated using Mann Whitney U test, based on comparison with homozygous *LEP* reference sequence (GG). Reference sequence: NM_000230.3(LEP).

* Statistical significance if *p* < 0.05.

## 4 Discussion

The results of this study provide insights into obesity-associated hematological alterations, demonstrating significant differences in blood cell populations between obese and control subjects. These findings contribute to the growing understanding of obesity’s systemic effects and identify potential *LEP* variants specific to the Saudi Arabian population.

The observed differences in demographic and anthropometric characteristics of the control and obese groups reflect the increased levels of obesity observed in females in Saudi Arabia. A systematic review analyzing 18 cross-sectional studies encompassing 96,000 individuals demonstrated higher obesity rates among Saudi women (36). This includes one study that found females to be twice as likely to be obese compared to males (37). Further analysis revealed that within obese groups, females had both a higher BMI and an increased proportion of class II and III obesity (38). This gender disparity in severe obesity has been consistently reported across multiple Saudi studies (39). Despite the demographic and anthropometric differences observed in this study, the hematological differences were consistent both across and within the obese and control groups.

The observed elevation in white blood cell (WBC) counts in obese individuals, compared to controls provides evidence for obesity-associated chronic inflammation in this population. Notably, findings of the current study demonstrate a progressive increase in WBC counts across obesity classes I, II, and III, suggesting a dose-dependent relationship between adiposity and systemic inflammation, although values remained within clinical reference ranges. These findings align with previous research, including a cross-sectional study by Takaya et al. (40) that identified significant correlations between BMI and both WBC and platelet counts.

The clinical significance of elevated WBC counts in obesity in the current study extends beyond their role as inflammatory markers, particularly in relation to type 2 diabetes (T2D) risk assessment. Previous longitudinal research has revealed that obese individuals with elevated WBC counts within the normal reference range, have a sixfold increased risk of T2D compared to controls (41). A critical WBC count threshold has been identified at 7 × 10^9^/L, above which the risk of T2D development increases significantly (41). Furthermore, elevated WBC counts appear to be an independent risk factor for T2D development, regardless of BMI status. A 9-year prospective study demonstrated that both obese and non-obese individuals with elevated WBC counts showed an increased risk of T2D compared to obese individuals with lower WBC counts, suggesting that inflammation, as measured by WBC count, may be a more crucial determinant of T2D risk than obesity alone (42).

The significant reduction in red blood cell indices (RBC count, hemoglobin, and hematocrit) in obese individuals provides new evidence for obesity-associated hematological alterations in the Saudi population. Hemoglobin differences may reflect sex-related physiological variation rather than obesity alone. The progressive decrease in these parameters across obesity classes strongly suggests a systematic effect of increasing adiposity on erythropoiesis. Previous research into the effects of obesity on red blood cell indices have revealed conflicting information. Whilst similar trends relating to reduction in red blood cell indices have been observed other studies have found no associations (43–46).

The mechanistic basis for these hematological alterations likely involves the complex interplay between adiposity, iron metabolism, and erythropoiesis. Obesity-associated chronic inflammation increases the production of hepcidin, a hormone involved in the regulation of iron homeostasis (47, 48). Elevated hepcidin levels lead to decreased iron absorption from the intestine, reduced iron release from macrophages and hepatocytes, resulting in lower serum iron concentrations and reduced iron availability for erythropoiesis. The dysregulation of iron homeostasis in obesity is characterized by impaired duodenal iron absorption, which is considered the pathophysiological hallmark of iron dysregulation in this condition (49). This impairment, coupled with elevated hepcidin levels, contributes to the development of iron deficiency and anemia in obese individuals, despite having adequate iron stores (49).

The genetic analysis of the current study identified two significant *LEP* variants with potential population-specific effects. The first variant, NM_00230.2:c.-39G > A (rs2167270) within exon 1 of the 5’-UTR region, identified in both the control and obese populations. Analysis of the genome aggregation database (gnomAD) (46) reveals a global variant allele frequency of 37.0% across combined exome and genome data, higher than the 12.8% frequency observed in the current study population (combined control and obese groups). Despite the high prevalence of this variant allele in the general population according to gnomAD data, the genetic association analysis of the current study demonstrated a significant relationship between the GA genotype of the c.-39G > A variant and increased obesity risk (OR: 2.48, 95% CI: 1.02–6.01).

This variant has been previously identified in cohort studies, with variable association with obesity. While some investigations have reported an increased obesity risk associated with this variant (50), others have found no significant relationship (29, 51), highlighting the complex nature of genetic contributions to obesity. However, the strong association of the c.-39G > A variant (rs2167270) and obesity risk identified in this study aligns with a similar study from the Arabic population, with a similar twofold risk increase identified (31). Furthermore, the GA variant phenotype has also previously been associated with increased risk of prediabetes (52). These associations suggest potential population-specific effects of this variant on obesity susceptibility.

The second variant NM_00230.2:c.280G > A (rs17151919) within exon 3 of *LEP*, was identified exclusively in the obese population, in four obese subjects. This variant, resulting in the non-conservative substitution of a valine amino acid with methionine in the leptin protein at position 94, p.(Val94Met), has previously been identified in association with low leptin levels in African ancestry and higher BMI (53). Yaghootkar et al., investigated this variant to determine the effects and influence on BMI and found that there was a significant association between the variant and increasing BMI between the ages of 3 and 7 (54). Furthermore, *in vitro* analysis of the variant found that there was lower secretion of leptin in cells expressing the variant allele, compared to the wild-type reference allele (54). Whilst this variant was not associated with any altered hematological parameters, the identification in the obese population suggests that this may be a genetic modifier associated with obesity or affecting untargeted metabolic profiling (55). Based on previous research, this may be through reduced leptin secretion, affecting the regulation of food intake and energy expenditure.

## 5 Limitations

Several limitations of this study warrant discussion and consideration. The cross-sectional design precludes the establishment of causal relationships. The disproportionate representation of females in the current study population, while reflecting documented gender disparities in Saudi Arabian obesity rates, may limit the generalizability of these findings across genders. Hemoglobin differences may reflect sex-related physiological variation rather than obesity alone. Additionally, the sample size may have limited the ability to detect subtle genetic effects, particularly for homozygous variant genotype for c.-39G > A, identified in five control and five obese individuals and the associations in obesity and WBC counts. We emphasize the need for larger cohorts and validation studies. In addition, whilst the c.-39G > A variant was associated with higher WBC counts, suggestive of a link to altered inflammatory response, the study would have benefited from direct measurement of pro-inflammatory markers to better characterize the relationship between obesity and low-grade inflammation.

## 6 Conclusion

In conclusion, this study provides a comprehensive analysis of the relationships between obesity, hematological parameters, and genetic variations in *LEP* within a Saudi population. The identification of progressive hematological changes across obesity classes, coupled with population-specific genetic variants, suggests novel mechanisms linking adiposity to systemic physiological alterations. Future studies with sex-matched cohorts or adjusted analyses are required to validate these findings. These findings have important clinical implications for healthcare in Saudi Arabia, particularly in the context of the country’s high obesity prevalence. The demonstrated relationship between WBC counts and obesity, coupled with known associations with T2D risk and the progressive alterations in hematological parameters across obesity classes identified in this study highlight possible benefit of regular hematological screening for monitoring of obese individuals. The identification of *LEP* gene variants associated with obesity risk and altered WBC counts highlights the potential for personalized medicine approaches, which could help identify individuals at higher risk for obesity-related complications. Future research should focus on larger-scale genetic studies in KSA and other Arabic populations to better understand population-specific effects of *LEP* variants. Such research could ultimately lead to more personalized approaches to obesity management in the Saudi population, considering both genetic factors and regional characteristics, providing more effective management strategies.

## Data Availability

The original contributions presented in this study are included in the article/supplementary material, further inquiries can be directed to the corresponding author.

## References

[1] Daghestani M Daghestani M Daghistani M Bjørklund G Chirumbolo S Warsy A. The influence of the rs1137101 genotypes of leptin receptor gene on the demographic and metabolic profile of normal Saudi females and those suffering from polycystic ovarian syndrome. *BMC Womens Health.* (2019) 19:10. 10.1186/s12905-018-0706-x 30635060 PMC6329086

[2] NCD Risk Factor Collaboration (NCD-RISC). Worldwide trends in underweight and obesity from 1990 to 2022: a pooled analysis of 3663 population-representative studies with 222 million children, adolescents, and adults. *Lancet.* (2024) 403:1027–50. 10.1016/S0140-6736(23)02750-2 38432237 PMC7615769

[3] Okunogbe A Nugent R Spencer G Powis J Ralston J Wilding J. Economic impacts of overweight and obesity: current and future estimates for 161 countries. *BMJ Glob Health.* (2022) 7:e009773. 10.1136/bmjgh-2022-009773 36130777 PMC9494015

[4] Althumiri N Basyouni M AlMousa N AlJuwaysim M Almubark R BinDhim N et al. Obesity in Saudi Arabia in 2020: prevalence, distribution, and its current association with various health conditions. *Healthcare.* (2021) 9:311. 10.3390/healthcare9030311 33799725 PMC7999834

[5] Moradi-Lakeh M El Bcheraoui C Afshin A Daoud F AlMazroa M Al Saeedi M et al. Diet in Saudi Arabia: findings from a nationally representative survey. *Public Health Nutr.* (2017) 20:1075–81. 10.1017/S1368980016003141 27974061 PMC10261507

[6] Malkin J Baid D Alsukait R Alghaith T Alluhidan M Alabdulkarim H et al. The economic burden of overweight and obesity in Saudi Arabia. *PLoS One.* (2022) 17:e0264993. 10.1371/journal.pone.0264993 35259190 PMC8903282

[7] Coker T Saxton J Retat L Alswat K Alghnam S Al-Raddadi R et al. The future health and economic burden of obesity-attributable type 2 diabetes and liver disease among the working-age population in Saudi Arabia. *PLoS One.* (2022) 17:e0271108. 10.1371/journal.pone.0271108 35834577 PMC9282435

[8] Kim J Kim J Jo M Cho E Ahn S Kwon Y et al. The roles and associated mechanisms of adipokines in development of metabolic syndrome. *Molecules.* (2022) 27:334. 10.3390/molecules27020334 35056647 PMC8781412

[9] Clemente-Suárez V Redondo-Flórez L Beltrán-Velasco A Martín-Rodríguez A Martínez-Guardado I Navarro-Jiménez E et al. The role of adipokines in health and disease. *Biomedicines.* (2023) 11:1290. 10.3390/biomedicines11051290 37238961 PMC10216288

[10] Ellulu M Patimah I Khaza’ai H Rahmat A Abed Y. Obesity and inflammation: the linking mechanism and the complications. *Arch Med Sci.* (2017) 13:851–63. 10.5114/aoms.2016.58928 28721154 PMC5507106

[11] Dandona P Aljada A Bandyopadhyay A. Inflammation: the link between insulin resistance, obesity and diabetes. *Trends Immunol.* (2004) 25:4–7. 10.1016/j.it.2003.10.013 14698276

[12] Kirichenko T Markina Y Bogatyreva A Tolstik T Varaeva Y Starodubova A. The role of adipokines in inflammatory mechanisms of obesity. *Int J Mol Sci.* (2022) 23:14982. 10.3390/ijms232314982 36499312 PMC9740598

[13] Kotb A El Fakih R Hanbali A Hawsawi Y Alfraih F Hashmi S et al. Philadelphia-like acute lymphoblastic leukemia: diagnostic dilemma and management perspectives. *Exp Hematol.* (2018) 67:1–9. 10.1016/j.exphem.2018.07.007 30075295

[14] Masood B Moorthy M. Causes of obesity: a review. *Clin Med.* (2023) 23:284–91. 10.7861/clinmed.2023-0168 37524429 PMC10541056

[15] Al-Amer O Hawasawi Y Oyouni A Alshehri M Alasmari A Alzahrani O et al. Study the association of transmembrane serine protease 6 gene polymorphisms with iron deficiency status in Saudi Arabia. *Gene.* (2020) 751:144767. 10.1016/j.gene.2020.144767 32422234

[16] Rath S Hawsawi Y Alzahrani F Khan M. Epigenetic regulation of inflammation: the metabolomics connection. *Semin Cell Dev Biol.* (2024) 154:355–63. 10.1016/j.semcdb.2022.09.008 36127262

[17] Speliotes E Willer C Berndt S Monda K Thorleifsson G Jackson A et al. Association analyses of 249,796 individuals reveal 18 new loci associated with body mass index. *Nat Genet.* (2010) 42:937–48. 10.1038/ng.686 20935630 PMC3014648

[18] Locke A Kahali B Berndt S Justice A Pers T Day F et al. Genetic studies of body mass index yield new insights for obesity biology. *Nature.* (2015) 518:197–206. 10.1038/nature14177 25673413 PMC4382211

[19] Ang M Takeuchi F Kato N. Deciphering the genetic landscape of obesity: a data-driven approach to identifying plausible causal genes and therapeutic targets. *J Hum Genet.* (2023) 68:823–33. 10.1038/s10038-023-01189-3 37620670 PMC10678330

[20] Al-Daghri N Al-Attas O Al-Rubeaan K Mohieldin M Al-Katari M Jones A et al. Serum leptin and its relation to anthropometric measures of obesity in pre-diabetic Saudis. *Cardiovasc Diabetol.* (2007) 6:18. 10.1186/1475-2840-6-18 17617917 PMC1933413

[21] Semlali A Almutairi M Rouabhia M Reddy Parine N Al Amri A Al-Numair N et al. Novel sequence variants in the TLR6 gene associated with advanced breast cancer risk in the Saudi Arabian population. *PLoS One.* (2018) 13:e0203376. 10.1371/journal.pone.0203376 30388713 PMC6214682

[22] Alzahrani O Mir R Alatwi H Hawsawi Y Alharbi A Alessa A et al. Potential impact of PI3K-AKT signaling pathway genes, KLF-14, MDM4, miRNAs 27a, miRNA-196a genetic alterations in the predisposition and progression of breast cancer patients. *Cancers.* (2023) 15:1281. 10.3390/cancers15041281 36831624 PMC9954638

[23] Alanazi I Shaik J Parine N Azzam N Alharbi O Hawsawi Y et al. Association of HER1 and HER2 gene variants in the predisposition of colorectal cancer. *J Oncol.* (2021) 2021:6180337. 10.1155/2021/6180337 34721579 PMC8553481

[24] Farooqi I Wangensteen T Collins S Kimber W Matarese G Keogh J et al. Clinical and molecular genetic spectrum of congenital deficiency of the leptin receptor. *N Engl J Med.* (2007) 356:237–47. 10.1056/NEJMoa063988 17229951 PMC2670197

[25] Paz-Filho G Mastronardi C Delibasi T Wong M Licinio J. Congenital leptin deficiency: diagnosis and effects of leptin replacement therapy. *Arq Bras Endocrinol Metabol.* (2010) 54:690–7. 10.1590/s0004-27302010000800005 21340154 PMC4286252

[26] Nordang G Busk ØL Tveten K Hanevik HI Fell AKM Hjelmesæth J et al. Next-generation sequencing of the monogenic obesity genes LEP, LEPR, MC4R, PCSK1 and POMC in a Norwegian cohort of patients with morbid obesity and normal weight controls. *Mol Genet Metab.* (2017) 121:51–6. 10.1016/j.ymgme.2017.03.007 28377240

[27] Fan S Say Y. Leptin and leptin receptor gene polymorphisms and their association with plasma leptin levels and obesity in a multi-ethnic Malaysian suburban population. *J Physiol Anthropol.* (2014) 33:15. 10.1186/1880-6805-33-15 24947733 PMC4073586

[28] Saeed S Bonnefond A Manzoor J Shabbir F Ayesha H Philippe J et al. Genetic variants in LEP, LEPR, and MC4R explain 30% of severe obesity in children from a consanguineous population. *Obesity.* (2015) 23:1687–95. 10.1002/oby.21142 26179253

[29] Ortega F Camberos A Arredondo M Magallanes N Meraz EA. LEP (G2548A-G19A) and ADIPOQ (T45G-G276T) gene polymorphisms are associated with markers for metabolic syndrome. *Diabetol Metab Syndr.* (2023) 15:237. 10.1186/s13098-023-01215-6 37978555 PMC10656912

[30] Younes S Ibrahim A Al-Jurf R Zayed H. Genetic polymorphisms associated with obesity in the Arab world: a systematic review. *Int J Obes.* (2021) 45:1899–913. 10.1038/s41366-021-00867-6 34131278 PMC8380539

[31] Bains V Kaur H Badaruddoza B. Association analysis of polymorphisms in LEP (rs7799039 and rs2167270) and LEPR (rs1137101) gene towards the development of type 2 diabetes in North Indian Punjabi population. *Gene.* (2020) 754:144846. 10.1016/j.gene.2020.144846 32512158

[32] Martins M Trujillo J Freitas-Vilela A Farias D Rosado E Struchiner C et al. Associations between obesity candidate gene polymorphisms (fat mass and obesity-associated (FTO), melanocortin-4 receptor (MC4R), leptin (LEP) and leptin receptor (LEPR)) and dietary intake in pregnant women. *Br J Nutr.* (2018) 120:454–63. 10.1017/S0007114518001423 29893663

[33] Alrefaei A Hawsawi Y Almaleki D Alafif T Alzahrani F Bakhrebah M. Genetic data sharing and artificial intelligence in the era of personalized medicine based on a cross-sectional analysis of the Saudi human genome program. *Sci Rep.* (2022) 12:1405. 10.1038/s41598-022-05296-7 35082362 PMC8791994

[34] Morales J Pujar S Loveland J Astashyn A Bennett R Berry A et al. A joint NCBI and EMBL-EBI transcript set for clinical genomics and research. *Nature.* (2022) 604:310–5. 10.1038/s41586-022-04558-8 35388217 PMC9007741

[35] den Dunnen J Dalgleish R Maglott D Hart R Greenblatt M McGowan-Jordan J et al. HGVS recommendations for the description of sequence variants: 2016 update. *Hum Mutat.* (2016) 37:564–9. 10.1002/humu.22981 26931183

[36] Salem V AlHusseini N Abdul Razack H Naoum A Sims O Alqahtani S. Prevalence, risk factors, and interventions for obesity in Saudi Arabia: a systematic review. *Obes Rev.* (2022) 23:e13448. 10.1111/obr.13448 35338558 PMC9287009

[37] Garawi F Ploubidis G Devries K Al-Hamdan N Uauy R. Do routinely measured risk factors for obesity explain the sex gap in its prevalence? Observations from Saudi Arabia. *BMC Public Health.* (2015) 15:254. 10.1186/s12889-015-1608-6 25848853 PMC4371623

[38] Almubark R Alqahtani S Isnani A Alqarni A Shams M Yahia M et al. Gender differences in the attitudes and management of people with obesity in Saudi Arabia: data from the ACTION-IO study. *Risk Manag Healthc Policy.* (2022) 15:1179–88. 10.2147/RMHP.S346206 35685203 PMC9172923

[39] Alhabib K Batais M Almigbal T Alshamiri M Altaradi H Rangarajan S et al. Demographic, behavioral, and cardiovascular disease risk factors in the Saudi population: results from the Prospective Urban rural epidemiology study (PURE-Saudi). *BMC Public Health.* (2020) 20:1213. 10.1186/s12889-020-09298-w 32770968 PMC7414714

[40] Takaya J Tanabe Y Nomura N Minami M Onuma C Yamagishi M et al. Platelet and white blood cell counts correlate with leptin and body mass index in Japanese adolescents. *Clin Pediatr Endocrinol.* (2024) 33:207–13. 10.1297/cpe.2024-0045 39359671 PMC11442701

[41] Twig G Afek A Shamiss A Derazne E Tzur D Gordon B et al. White blood cells count and incidence of type 2 diabetes in young men. *Diabetes Care.* (2013) 36:276–82. 10.2337/dc11-2298 22961572 PMC3554323

[42] Gu Y Hu K Huang Y Zhang Q Liu L Meng G et al. White blood cells count as an indicator to identify whether obesity leads to increased risk of type 2 diabetes. *Diabetes Res Clin Pract.* (2018) 141:140–7. 10.1016/j.diabres.2018.04.041 29730387

[43] Bagni U Luiz R Veiga G. Overweight is associated with low hemoglobin levels in adolescent girls. *Obes Res Clin Pract.* (2013) 7:e218–29. 10.1016/j.orcp.2011.12.004 23697591

[44] Ghadiri-Anari A Nazemian N Vahedian-Ardakani H. Association of body mass index with hemoglobin concentration and iron parameters in Iranian population. *ISRN Hematol.* (2014) 2014:525312. 10.1155/2014/525312 24665367 PMC3934448

[45] Mehdad S Benaich S Hamdouchi A Bouhaddou N Azlaf M Menchawy I et al. Association between overweight and anemia in Moroccan adolescents: a cross-sectional study. *Pan Afr Med J.* (2022) 41:156. 10.11604/pamj.2022.41.156.20927 35573439 PMC9058990

[46] Chen S Francioli L Goodrich J Collins R Kanai M Wang Q et al. A genomic mutational constraint map using variation in 76,156 human genomes. *Nature*. (2024) 625:92–100. 10.1038/s41586-023-06045-0 38057664 PMC11629659

[47] Alshwaiyat N Ahmad A Wan Hassan W Al-Jamal H. Association between obesity and iron deficiency (Review). *Exp Ther Med.* (2021) 22:1268. 10.3892/etm.2021.10703 34594405 PMC8456489

[48] Al-Amer O Oyouni A Alshehri M Alasmari A Alzahrani O Aljohani S et al. Association of SNPs within TMPRSS6 and BMP2 genes with iron deficiency status in Saudi Arabia. *PLoS One.* (2021) 16:e0257895. 10.1371/journal.pone.0257895 34780475 PMC8592490

[49] Aigner E Feldman A Datz C. Obesity as an emerging risk factor for iron deficiency. *Nutrients.* (2014) 6:3587–600. 10.3390/nu6093587 25215659 PMC4179177

[50] Araujo Junior A de Azevedo G Moliterno L Tavares R Cardoso J de Souza G et al. Association of polymorphism in leptin receptor gene with susceptibility of adolescent idiopathic scoliosis. *Eur Spine J.* (2024) 33:646–54. 10.1007/s00586-023-07955-3 37801129

[51] Berezina A Belyaeva O Berkovich O Baranova E Karonova T Bazhenova E et al. Prevalence, risk factors, and genetic traits in metabolically healthy and unhealthy obese individuals. *Biomed Res Int.* (2015) 2015:548734. 10.1155/2015/548734 26504811 PMC4609360

[52] Dasgupta S Salman M Siddalingaiah L Lakshmi G Xaviour D Sreenath J. Genetic variants in leptin: determinants of obesity and leptin levels in South Indian population. *Adipocyte.* (2015) 4:135–40. 10.4161/21623945.2014.975538 26167411 PMC4496968

[53] Friedlander Y Li G Fornage M Williams O Lewis C Schreiner P et al. Candidate molecular pathway genes related to appetite regulatory neural network, adipocyte homeostasis and obesity: results from the CARDIA Study. *Ann Hum Genet.* (2010) 74:387–98. 10.1111/j.1469-1809.2010.00596.x 20642810 PMC2945878

[54] Yaghootkar H Zhang Y Spracklen C Karaderi T Huang L Bradfield J et al. Genetic studies of leptin concentrations implicate leptin in the regulation of early adiposity. *Diabetes.* (2020) 69:2806–18. 10.2337/db20-0070 32917775 PMC7679778

[55] Alzahrani F Shait Mohammed M Alkarim S Azhar E El-Magd M Hawsawi Y et al. Untargeted metabolic profiling of extracellular vesicles of SARS-CoV-2-infected patients shows presence of potent anti-inflammatory metabolites. *Int J Mol Sci.* (2021) 22:10467. 10.3390/ijms221910467 34638812 PMC8509011

